# Rapid reviews for rapid decision-making during the coronavirus disease (COVID-19) pandemic, Norway, 2020

**DOI:** 10.2807/1560-7917.ES.2020.25.19.2000687

**Published:** 2020-05-14

**Authors:** Atle Fretheim, Kjetil G Brurberg, Frode Forland

**Affiliations:** 1 Norwegian Institute of Public Health, Oslo, Norway

**Keywords:** systematic reviews, rapid evidence synthesis, Covid-19, decision support

## Abstract

In response to urgent needs for updated evidence for decision-making on various aspects related to coronavirus disease (COVID-19), the Norwegian Institute of Public Health established a rapid review team. Using simplified processes and shortcuts, this team produces summary reviews on request within 1–3 days that inform advice provided by the institute. All reviews are published with explicit messages about the risk of overlooking key evidence or making misguided judgements by using such rapid processes.

When the coronavirus disease (COVID-19) pandemic, caused by the severe acute respiratory syndrome coronavirus 2 (SARS-CoV-2) started to unfold in Europe at the beginning of 2020 [1], the Norwegian Institute of Public Health (NIPH), as other public health agencies, mobilised forces and implemented contingency plans. NIPH experts soon faced difficulties when trying to fulfil their mission to provide evidence-informed guidance to the public, health services and policymakers, as updated reviews of research findings on COVID-19 related topics were not readily available, e.g. on the transmissibility of the virus or identifying groups at particular risk of severe disease.

While keeping up with scientific developments is challenging also under normal circumstances, the combination of uncertainties in dealing with a novel virus and a huge outpour of research papers dealing with COVID-19, many of them not peer-reviewed, made it particularly challenging to provide evidence-informed guidance, either to the public, health services or policymakers.

## Rapid review team

Evidence-informed decision-making should rely on the best available evidence, typically in the form of systematic reviews [2]. In a systematic review, relevant research findings on a given topic, guided by specific research questions, are identified, appraised and summarised through a transparent and scientifically sound process [3]. Since COVID-19 is a novel disease [1], at its onset there were very few systematic reviews to base decisions on. In addition, systematic reviews on COVID-19-related topics were likely to become rapidly outdated, given the large number of new study reports published daily.

Scarcity of evidence and shortage of time pose specific challenges, for evidence-informed decision-making [4]. To meet the urgent need for updated evidence, NIPH set up a rapid review team of 2–3 researchers and an information specialist. All team members had wide experience in conducting systematic reviews and health technology assessments, but no specific expertise in epidemics or infectious diseases. The NIPH’s task force for managing the national response to the pandemic, selected and prioritised topics for rapid review through an informal process, based on requests from stakeholders and the perceived needs.

After we established the team on 20 March 2020, it immediately started working on the following topics: the risk of airborne transmission, the role of children in the spread of disease, the relationship between age, comorbidity and disease severity, immunity to COVID-19, and transmission via surfaces. By 19 April, we had published eight rapid reviews, which were made available on the institute’s website [5-12].

Since speed was imperative, the rapid review team intended to simplify their normal systematic review production process to the extent that reviews could be prepared in a matter of days, instead of the several months usually needed to prepare a full-fledged systematic review.

## References search

Normally, a rapid review will rely on existing systematic reviews in order to be timely prepared [13]. This was only partly possible in the present situation, since most questions were specific for COVID-19 and indirect evidence from studies on other viral diseases, e.g. influenza or severe acute respiratory syndrome (SARS), was of limited interest.

An advantage, in terms of speed, was that we could restrict our literature searches to COVID-19 publications, thus limiting each search on a specific aspect to publications from the last few months, and use COVID-19-specific search engines, e.g. the United States National Library of Medicine’s new literature hub, LitCovid [14]. This made the number of hits manageable. On the other hand, we felt obliged to search for non-peer reviewed reports from preprint servers such as medRxiv. This increased the workload substantially, as this meant having to assess a large number of additional titles and reports, and because the quality of these manuscripts proved highly variable.

The team members individually screened potentially relevant titles, i.e. not in duplicate or with double-checking, as would be the normal practice for systematic reviews. Similarly, each team member assessed the relevance and validity of each study, and checklists or other formal approaches were not used. Meta-analyses were not conducted, and findings from the included studies were summarised narratively. This process entailed some degree of judgement, especially concerning which findings to emphasise, or not. All results and conclusions were discussed by two team members. We also had a rapid peer review process, which meant that at least two content experts – often the commissionaires – reviewed the manuscript.

## Transparency about limitations

Our rapid review process entailed many shortcuts compared with the standard systematic-review approach – probably also in comparison with most other rapid reviews. To make the inherent risks explicit, we always included a description of the method as well as the following statement: “*In the current situation, there is an urgent need for identifying the most important evidence quickly. Hence, we opted for this rapid approach despite an inherent risk of overlooking key evidence or making misguided judgements*.”

## A dynamic field

Given the infancy of the field and the large volume of COVID-19 related research, the rapid reviews need regular updates to remain relevant. Until now, we have updated one rapid review, which serves as an example of the evolving evidence base: We published the first rapid review on the relationship between age, comorbidity and disease severity on 26 March [7]. At that time, we only identified three studies with the multivariate analyses needed to distinguish between the effect of age and of comorbidities. Three weeks later, in our updated review [12], we were able to include 12 studies with multivariate analyses. Our comment in the discussion section expresses the frustration that many may feel when assessing large amounts of COVID-19 research articles, often of questionable quality: “*There seems to be an abundance of publications reporting on small samples of patients, with simple univariate analyses of risk factors for severe COVID-19. In our view, such studies contribute little to improving our understanding of the importance of various risk factors. We encourage medical journals to refuse publication of additional research papers with small sample sizes, and to require multivariate analysis.*”

## Conclusions

Being a national public health institute with responsibilities for infectious diseases prevention and response, as well as having the role as the national centre for evidence-based healthcare, meant that the competence and tools to develop the rapid reviews were available within the organisation. This allowed to establish fast and efficient collaborations across relevant units. The rapid reviews are highly valued and seen as key contributions to the evidence that informs advice provided by the institute. The rapid reviews have also been widely cited in the media.

Our expectation for the upcoming weeks and months is that the methodology of our rapid reviews will evolve towards the more thorough and standardised systematic review processes. We acknowledge that there are initiatives parallel to ours, taken by other institutions, using slightly different methodologies and published in different languages. With this current report, we hope to share methodologies and results and to prevent redundancy and overlap. [15-19]. Given the large volume of new publications on COVID-19 with highly variable quality, we have no doubt that systematic reviews will play a key role in informing an evidence-based management of the ongoing pandemic.

As of 12 May, all our COVID-19 rapid reviews are available in English versions: https://www.fhi.no/en/sys/news/?blockId=90733&ownerPage=45271&language=en

Another initiative at our institute is the creation of a living map of available evidence for COVID-19 – a collaborative effort with McMaster University and the Cochrane Canada Centre – to ease the work for reviewers internationally: https://www.fhi.no/en/qk/systematic-reviews-hta/map/
